# Exploring Competencies That Prepare Occupational Therapy Students for Acute Mental Health Practice: A Document Analysis

**DOI:** 10.1155/oti/5063621

**Published:** 2026-05-26

**Authors:** Phakeme Z. Mkhize, December M. Mpanza, Deshini Naidoo

**Affiliations:** ^1^ Discipline of Occupational Therapy, School of Health Sciences, College of Health Sciences, University of KwaZulu-Natal, Durban, South Africa, ukzn.ac.za

**Keywords:** acute mental health, competency framework, curriculum development, document analysis, occupational therapy, South Africa

## Abstract

Occupational therapy graduates in South Africa are increasingly required to practice in acute mental health settings, yet the extent to which current curricula prepare them adequately remains underexplored. This study examined the competencies required for acute mental health occupational therapy practice through a comparative document analysis of four national and international frameworks: the WFOT Minimum Standards (2016), HPCSA Minimum Standards (2024), WHO Recommendations for Occupational Therapy Practice in Mental Health (2024), and WHO mhGAP Intervention Guide (2016), mapped against the University of KwaZulu‐Natal (UKZN) exit‐level outcomes (2024). Seven core competency domains were identified across the frameworks. The UKZN outcomes demonstrated strong alignment in cultural competence and interdisciplinary collaboration, partial alignment in recovery‐oriented practice, assessment skills, therapeutic use of self, and professional resilience, and confirmed gaps in condition‐specific knowledge, risk assessment, trauma‐informed care, crisis intervention, and transition planning. These findings demonstrate that acute mental health competency preparation cannot be achieved through alignment with any single regulatory document, including the HPCSA framework. This therefore calls for both urgent curriculum reform and strengthening of accreditation standards across all South African occupational therapy programs.

## 1. Introduction

Mental health disorders affect approximately 970 million people globally, with low‐ and middle‐income countries bearing a disproportionate burden while having the fewest resources to respond [1, 2]. In South Africa, an estimated 16.5% of adults are affected, yet a 75% treatment gap persists, meaning that the majority of those who need care do not receive it [3]. This gap is particularly acute in public sector settings where occupational therapists are most likely to practice, emphasizing the urgency of equipping graduates with the specialized competencies required for acute mental health care [4].

South Africa′s policy architecture, anchored by the Mental Health Care Act No. Seventeen of 2002 [5] and the National Mental Health Policy Framework and Strategic Plan 2023–2030 [6], reflects an intent to integrate mental health into primary healthcare and to protect the rights of persons with mental disorders [6, 7]. However, implementation has been constrained by workforce shortages, resource limitations, and inadequate specialist services, with the result that acute inpatient units continue to carry a disproportionate share of unmet need [4, 8]. It is within this resource‐constrained yet high‐demand context that occupational therapy graduates must be equipped to practice competently in acute mental health settings from the outset of their careers.

Occupational therapy makes a vital contribution to mental health recovery through its focus on meaningful occupation, environmental adaptation, and psychosocial rehabilitation, a contribution grounded in well‐established occupational therapy models. The model of human occupation [9] provides a particularly relevant framework, explaining how volition, habituation, and performance capacity interact with environmental contexts to shape occupational participation. In acute mental health settings, psychiatric symptoms disrupt all three components simultaneously: volition is compromised by hopelessness and negative symptoms; habitual routines collapse during inpatient admission; and performance capacity is affected by both the disorder itself and its pharmacological management. The Canadian Model of Occupational Performance and Engagement [10] with its explicit emphasis on spirituality as central to occupational identity and its person‐occupation‐environment framework, has been applied in South African occupational therapy education and practice, aligning with the UKZN program′s focus on contextually responsive, person‐centered care. In acute mental health settings, occupational therapists address functional impacts of psychiatric symptoms, develop coping strategies, facilitate meaningful activity engagement during hospitalization, and prepare clients for community reintegration [11, 12]. Evidence supports occupational therapy′s effectiveness in improving functioning, reducing relapse rates, and enhancing quality of life for people with serious mental illness [13]. However, a critical shortage of occupational therapists working in mental health exists globally despite the field′s historical roots in this area [14]. In South Africa, this shortage is particularly acute, with that only 5% of South African occupational therapists work in psychiatric facilities despite the significant need [15, 16]. Reasons include funding constraints, role ambiguity in multidisciplinary teams, and graduate preferences for other practice areas [17]. This workforce gap contributes significantly to the treatment gap for mental health services, particularly in resource‐constrained settings like KwaZulu‐Natal.

Occupational therapy education in Africa has evolved significantly since the first program was established at the University of the Witwatersrand in 1943 [18]. Currently, South Africa hosts eight occupational therapy education programs, all of which must meet the accreditation standards of both the World Federation of Occupational Therapists (WFOT) and the Health Professions Council of South Africa (HPCSA). Joubert [19] observed that African occupational therapy curricula face the challenge of balancing internationally derived competency standards with locally relevant approaches addressing the continent′s unique health priorities and contextual realities. For example, internationally derived competency frameworks may specify that occupational therapists conduct standardized individual assessments of occupational performance and develop person‐centered, goal‐directed intervention plans; yet in many South African public sector acute mental health settings, resource constraints, large caseloads, and collectivist community values necessitate adaptations such as group‐based assessments, family‐inclusive goal‐setting, and interventions that integrate cultural and spiritual dimensions of recovery that are not addressed in globally standardized tools. Mental health education specifically has undergone significant evolution, with Janse van Rensburg [20] noting a shift from primarily biomedical, institution‐based approaches toward recovery‐oriented, rights‐based, and community‐centered practice. However, Weir et al. [21] found that many South African occupational therapy curricula still dedicate less time to mental health practice than to physical rehabilitation, potentially contributing to the workforce shortage in this area.

Research indicates significant challenges in preparing graduates for acute mental health practice. Wimpenny and Lewis [22] and Lloyd et al. [23] found that newly qualified occupational therapists reported feeling underprepared for the realities of acute psychiatric settings, particularly regarding risk management and therapeutic interventions during psychiatric crises. This echoes international findings by Scanlan et al. [24], who identified acute mental health as an area where graduates consistently report lower confidence compared with other practice areas. Curricular challenges contributing to this gap include limited acute mental health fieldwork placements, with Golos and Tekuzener [25] documenting that in general, occupational therapy students receive on average only a few weeks of acute psychiatric placement throughout their four‐year degree. Additionally, Wimpenny et al. [26] identified that teaching about specialized mental health assessments and interventions is often constrained by limited faculty expertise and competing curriculum demands.

Educational approaches for acute mental health competency development have shown varying effectiveness. Problem‐based learning methodologies have demonstrated positive outcomes in developing clinical reasoning for complex psychiatric presentations, according to a South African study by Jay [27]. Similarly, Singano [28] found that simulation‐based education significantly improved students′ confidence in acute mental health assessment skills. However, research by De Jongh [29] indicated that these educational innovations remain inconsistently implemented across South African occupational therapy programs, with many still relying primarily on traditional didactic approaches for acute mental health content. Furthermore, graduate competency frameworks used by South African programs often lack sufficient detail regarding acute mental health practice specifically where competency statements tend to address mental health generically without differentiating the specialized skills required for acute inpatient settings [30–32].

Despite the availability of guiding frameworks, research consistently indicates that newly qualified South African occupational therapists feel underprepared for the realities of acute psychiatric practice [22, 24]. Competency statements across programs tend to address mental health generically, without differentiating the specialized knowledge, skills, and attitudes required for acute inpatient settings. This gap between what frameworks prescribe and what graduates experience in practice represents the central problem this study addresses [30–32]. Accordingly, this study is aimed at exploring the competencies required for occupational therapy practice in acute mental health settings through a comparative document analysis of key national and international frameworks, with a view to identifying curricular enhancements necessary to better prepare graduates for this demanding practice context.

## 2. Methodology

### 2.1. Study Context

This study was conducted at the University of KwaZulu‐Natal (UKZN), one of eight institutions offering occupational therapy education in South Africa. Situated in KwaZulu‐Natal province, the university serves a diverse population with significant mental health needs exacerbated by high rates of HIV/AIDS, substance abuse, and trauma‐related conditions [33]. UKZN′s Bachelor of Occupational Therapy is a 4‐year professional degree program established in 1981, with a current annual intake of approximately 46–50 students [34]. The curriculum includes dedicated mental health modules across all 4 years of study, with students completing an approximately 6–7 weeks acute mental health fieldwork placement in their fourth year at psychiatric hospitals within the province. This study specifically examined competencies for acute mental health practice, recognizing this as a critical area where occupational therapists make significant contributions despite ongoing workforce shortages.

### 2.2. Research Design

This study employed document analysis, a systematic procedure for reviewing and evaluating documents to elicit meaning, gain understanding, and develop empirical knowledge [35]. Document analysis is a recognized qualitative research methodology well‐suited to the comparison of formal competency frameworks, policy documents, and educational standards across different organizational and contextual perspectives. This design was selected because the research question: *What competencies do occupational therapy graduates require for acute mental health practice, and how does the UKZN programme address these?* The approach enabled systematic comparison without the limitations that would arise from relying solely on practitioner or educator self‐report.

### 2.3. Document Selection and Search Strategy

**Figure 1:**
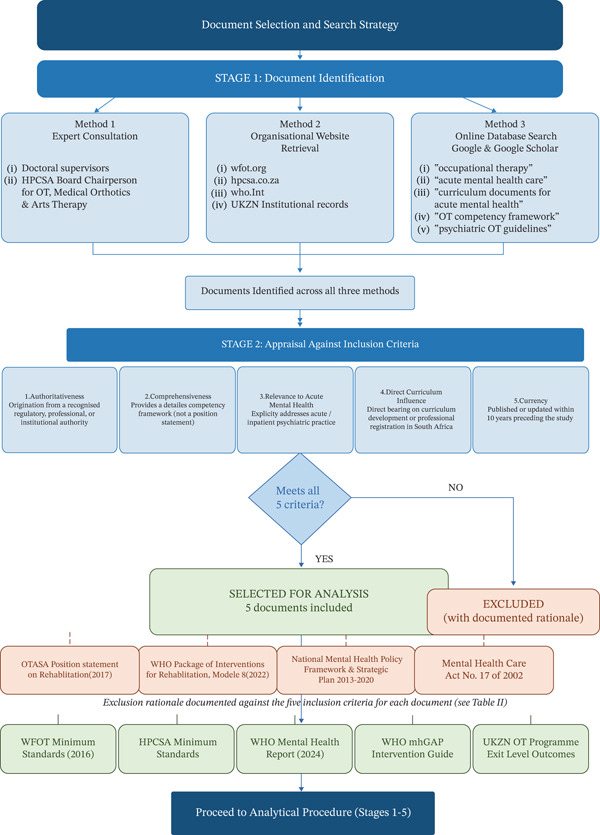
Flowchart of document selection and search strategy.

Document selection followed a purposive sampling strategy guided by the study′s aim to compare internationally recognized competency standards with the UKZN program′s exit‐level outcomes. The selection process was conducted in two stages: a scoping search phase and a critical appraisal and selection phase (Figure 1).

#### 2.3.1. Stage 1: Document Identification

Documents were identified through three complementary methods, used in combination to ensure that relevant authoritative sources were not overlooked.

i. Expert consultation: The first author′s doctoral supervisors were consulted at the outset of the study to assist in identifying which policy, regulatory, and professional documents would be most relevant to a competency analysis focused on acute mental health occupational therapy education. In addition, the first author directly contacted the Chairperson of the HPCSA Professional Board for Occupational Therapy, Medical Orthotics and Prosthetics, and Arts Therapy, who provided guidance on documents relevant to the regulation of occupational therapy education and practice in South Africa. This expert consultation ensured that the document identification process was grounded in current regulatory knowledge rather than relying solely on database searches
ii. Targeted retrieval from official organizational websites: Based on the researchers′ knowledge of the regulatory and professional landscape governing occupational therapy education in South Africa, the official websites of relevant professional and regulatory bodies were searched directly. These included the WFOT, HPCSA, the World Health Organization and the UKZN occupational therapy program′s institutional records. This approach, consistent with grey literature retrieval in document analysis research, was appropriate given that the documents required regulatory standards, professional frameworks, and educational outcome documents are typically not indexed in academic databases but are published directly by their authoring organizations
iii. Online database search: To identify any additional relevant frameworks or curriculum documents not captured through the above methods, the first author conducted searches on Google and Google Scholar using the following search terms: “occupational therapy,” “acute mental health care,” “curriculum documents for acute mental health,” “occupational therapy competency framework,” “mental health education standards,” “occupational therapy mental health curriculum,” “psychiatric occupational therapy guidelines,” and “mental health gap action programme.” These searches were conducted to determine whether any other authoritative competency frameworks or educational documents existed beyond those already identified through expert consultation and organizational website retrieval. The WHO Mental Health Gap Action Programme (mhGAP) Intervention Guide was identified through this search and subsequently appraised against the inclusion criteria, which it met. No other additional documents meeting the inclusion criteria were identified through this method. Documents identified through this database search were included if they met all five criteria described in Stage 2 below, and excluded if they failed to meet any single criterion. Key exclusion reasons applied to database search results included: position statements rather than competency frameworks (failing the comprehensiveness criterion); documents addressing rehabilitation broadly without acute mental health specificity (failing the relevance criterion); and documents predating the 10‐year currency window. Full inclusion and exclusion criteria and their application are detailed in Stage 2 and Tables 1 and 2

**Table 1:** Documents selected for analysis.

Document	Rationale for selection
**WFOT minimum standards for the education of occupational therapists**	This is the he authoritative international standard recognized by all occupational therapy programs worldwide. It provides a global benchmark for competency expectations, including specific requirements for mental health practice. Selected as the primary international reference point against which other frameworks were compared.
**HPCSA minimum standards for the education and training of occupational therapists**	This is the primary national regulatory document governing professional qualification and practice in South Africa. All South African occupational therapy programs, including UKZN, must align with these standards for accreditation purposes. Its regulatory authority makes it foundational for the South African curriculum development context.
**WHO recommendations for occupational therapy practice in mental health**	This document was developed specifically to address occupational therapy′s role in mental health services globally, this document articulates contemporary best‐practice competencies across service levels including acute care. It was selected for its detailed, occupation‐specific guidance and its direct relevance to the competency domains under investigation.
**WHO Mental Health Gap Action Programme (mhGAP) Intervention Guide**	This is a practical implementation guide for priority mental health conditions in low‐ and middle‐income countries (LMICs), directly relevant to the South African context. It provides condition‐specific intervention competencies, including for depression, psychosis, substance use, and self‐harm, and incorporates occupational perspectives within multidisciplinary frameworks, filling a contextually specific gap not addressed by other selected documents.
**UKZN occupational therapy program exit level outcomes**	This is the institutional competency framework governing the UKZN occupational therapy program and the primary object of this study′s gap analysis. It defines the knowledge, skills, and attitudes graduates are expected to demonstrate upon completion of the degree, and served as the comparator document against which all other frameworks were mapped.

**Table 2:** Documents considered but excluded from analysis.

Document	Reason for exclusion
OTASA Position Statement On Rehabilitation (2017) [36]	This document was excluded on two grounds: first, it constitutes a position statement rather than a comprehensive competency framework, and therefore does not meet the comprehensiveness criterion; second, its focus is on rehabilitation broadly rather than on acute mental health competencies specifically. It was determined to be supplementary to, rather than distinct from, the HPCSA and WFOT frameworks already included.
WHO Package Of Interventions For Rehabilitation, Module 8—Mental Health Conditions (2023) [37]	This document was excluded because its mental health module addresses only schizophrenia, failing to cover the broad spectrum of acute mental health conditions relevant to occupational therapy practice. It also lacks specificity regarding assessment, intervention, and discharge planning procedures in acute settings, providing insufficient depth for the competency comparison this study required.
National Mental Health Policy Framework and Strategic Plan 2013–2020 (Department of Health, Republic of South Africa) [6]	This is a systems‐level policy and planning document that sets service delivery targets and resource allocation priorities in SA. It contains no knowledge–skills–attitudes framework and does not specify practitioner‐level competencies, failing the comprehensiveness and direct curriculum influence criteria. Its influence on the UKZN program is indirect, mediated through the HPCSA framework already included. It was acknowledged in the literature review as an important contextual document but excluded as it operates at a systemic rather than individual practitioner competency level.
Mental Health Care Act No. 17 of 2002 (Republic of South Africa) [5]	This document is a National legislation that defines legal rights, consent procedures, admission categories, and governance structures for mental health care. It contains no practitioner competency content, failing the comprehensiveness criterion entirely. Legal compliance with the Act is an expected baseline of all professional practice already embedded within the HPCSA framework included in this study. It was acknowledged in the literature review as foundational context but excluded as legislation falls outside the analysis of individual practitioner competencies that meets this study′s aim

#### 2.3.2. Stage 2: Appraisal and Selection

Each identified document was appraised against five inclusion criteria: (1) authoritativeness: the document must originate from a recognized regulatory body, international health authority, or the institution under study; (2) comprehensiveness: the document must provide a detailed competency framework rather than a general position statement; (3) relevance to acute mental health: the document must explicitly or substantially address mental health knowledge, skills, and attitudes relevant to acute or inpatient psychiatric practice; (4) direct influence on curriculum: the document must have direct bearing on curriculum development or professional registration requirements for South African occupational therapy graduates; and (5) currency: the document must represent current standards, defined as having been published or most recently updated within the 10 years preceding the study. Following this appraisal, four documents met all inclusion criteria and were selected for analysis. The remaining documents were excluded; four of these exclusions are presented in Table 2 with the reasoning applied to each.

### 2.4. Analytical Procedure

The analysis followed a structured five‐stage procedure, consistent with Bowen′s [35] framework for document analysis. Each stage built directly on the preceding one, moving from individual document reading through to comparative synthesis and gap identification.

#### 2.4.1. Stage 1: Individual Document Review and Competency Extraction

Each of the four guiding documents (WFOT, HPCSA, WHO Recommendations, and WHO mhGAP) was read in full by the first author to understand its overall structure, purpose, and contextual grounding. Acute mental health competencies were then systematically extracted from each document and organized using the knowledge–skills–attitudes (KSA) framework commonly employed in professional and health sciences education. It is important to note that not all documents used explicit KSA language or structured their content according to this framework. Where a document presented competencies in an alternative format, for example, as clinical protocols, intervention algorithms, or general principles of care, the first author synthesized the competency content and mapped it onto the KSA categories based on the nature of what was described: declarative content (what a practitioner needs to *know*) was mapped to knowledge; procedural or applied content (what a practitioner needs to *do*) was mapped to skills; and dispositional or professional orientation content (how a practitioner needs to *approach* their work) was mapped to attitudes. This interpretive mapping process was discussed with the supervisors at key points to ensure consistency and to challenge assumptions in the categorization. Each extracted competency was recorded with its corresponding page number and section reference within the source document to ensure traceability. These extractions constitute Tables 3, 4, 5, and 6 below which present the full extraction for each guiding document.

**Table 3:** Competency extraction: WFOT Minimum Standards for the Education of Occupational Therapists [38].

Competency area	Knowledge	Skills	Attitudes	Source page(s)
Therapeutic use of self	• Theoretical understanding of therapeutic relationships • Communication theories for psychological distress • Professional boundaries in acute settings • Trauma‐informed approaches	• Establishing rapport with clients in acute distress • Advanced communication skills • Maintaining therapeutic presence during crises • De‐escalation techniques • Adapting communication style	• Genuine empathy and positive regard • Respect for client autonomy • Nonjudgmental stance • Self‐awareness • Openness to challenging relationships	pp. 17–18, 32
Assessment Skills	• OT assessment frameworks for mental health • Standardized tools for psychiatric populations • Impact of conditions on occupational performance • Risk assessment protocols	• Selecting appropriate assessment methods • Conducting evaluations in time‐limited settings • Observational assessment skills • Interpreting findings • Clear documentation	• Respect for client perspective • Flexibility in approaches • Recognition of client expertise • Commitment to holistic assessment • Thoroughness	pp. 22–23, 38
Intervention planning	• Evidence‐based interventions for acute mental health • Occupation‐based approaches for acute care • Relationship between occupation and recovery • Group and individual approaches • Environmental modifications	• Developing targeted intervention plans • Adapting to acute care constraints • Implementing therapeutic activities • Grading activities based on mental state • Teaching coping strategies	• Creativity within limitations • Flexibility in adjusting plans • Commitment to occupation‐focused practice • Perseverance with reluctant clients • Openness to feedback	pp. 24–26, 40–41
Clinical reasoning	• Clinical reasoning models for mental health • Decision‐making frameworks for priority‐setting • Contextual factors in clinical decisions • Evidence integration processes	• Applying reasoning strategies in fast‐paced environments • Making sound clinical judgments • Analyzing complex performance issues • Articulating reasoning processes • Reflective practice	• Critical thinking • Willingness to question assumptions • Openness to revising judgments • Comfort with uncertainty • Commitment to evidence‐informed practice	pp. 19–20, 33–34
Mental health conditions	• Acute psychiatric conditions and symptoms • Psychopharmacology effects on performance • Common comorbidities • Occupational impact of conditions • Recovery processes and trajectories	• Recognizing various condition manifestations • Adapting practice based on diagnosis • Identifying occupational disruptions • Monitoring mental state changes • Client education from occupational perspective	• Nonstigmatizing approach • Recognition of person beyond diagnosis • Respect for diverse experiences • Willingness to learn from clients • Optimism about recovery potential	pp. 28–29, 43
Risk assessment	• Suicide risk factors and warning signs • Violence risk assessment frameworks • Self‐harm behaviors and interventions • Safety protocols • Documentation requirements	• Conducting suicide/self‐harm assessments • Implementing safety plans • De‐escalation and crisis intervention • Appropriate risk documentation • Communicating risk to team members	• Vigilance regarding safety • Balancing risk management with autonomy • Maintaining therapeutic optimism • Willingness for difficult conversations • Commitment to least restrictive approaches	pp. 30, 44–45
Interdisciplinary collaboration	• Multidisciplinary team structures • Roles of other health professionals • Referral processes and pathways • Team communication strategies • OT′s unique contribution	• Communicating OT perspective • Interprofessional collaboration • Negotiating role boundaries • Advocating for occupational needs • Documentation supporting team communication	• Respect for other disciplines • Knowledge sharing and learning • Confidence in articulating OT value • Collaborative problem‐solving • Commitment to team communication	pp. 35–36, 48
Recovery‐oriented practice	• Recovery principles in acute settings • Strengths‐based approaches • Occupation′s role in recovery • Hope‐inspiring strategies • Personal and social recovery dimensions	• Identifying client strengths despite symptoms • Fostering hope about recovery • Supporting autonomy in restricted environments • Identifying meaningful occupations • Reconnecting with valued roles	• Belief in recovery possibilities • Recognition of client expertise • Commitment to person‐centered approaches • Hope‐inspiring stance • Respect for diverse recovery pathways	pp. 27, 42
Ethical practice	• Ethical principles in mental health • Legal frameworks for involuntary treatment • Capacity assessment and informed consent • Common ethical dilemmas • Human rights frameworks	• Analyzing ethical dilemmas • Supporting decision‐making • Balancing care with autonomy • Advocating for ethical treatment • Documenting ethical decisions	• Commitment to ethical standards • Respect for client rights • Willingness to address challenges • Courage to advocate • Reflective ethical decision‐making	pp. 14–15, 31
Cultural competence	• Cultural influences on mental health • Diverse beliefs about mental health • Impact of culture on occupational values • Culturally responsive approaches • Effects of discrimination on mental health	• Conducting culturally sensitive assessments • Adapting interventions to cultural contexts • Working with interpreters • Recognizing cultural influences on occupation • Advocating for culturally appropriate services	• Cultural humility and learning • Respect for diverse perspectives • Recognition of own cultural biases • Openness to alternative explanatory models • Addressing cultural power imbalances	pp. 16, 37

**Table 4:** Competency extraction: HPCSA Minimum Standards for the Education and Training of Occupational Therapists.

Knowledge competencies	Skills competencies	Attitude competencies
**Section 3.4: Knowledge of Mental Health Conditions and Psychopharmacology**		
• Knowledge of psychiatric conditions (DSM/ICD) (p.14–15, Section 3.4) • Understanding of aetiology and clinical picture (p.15, Section 3.4) • Knowledge of prevention, diagnosis, management (p.15, Section 3.4) • Understanding precautions for various conditions (p.15, Section 3.4) • Knowledge of psychopharmacology effects (implied in p.15, Section 3.4)	• Conducting holistic assessments of mental health′s impact on occupation (p.19, Section 4.5.1) • Screening for mental health risk factors (p.19, Section 4.5.1) • Applying perceptual testing techniques (p.20, Section 4.5.1)	• Professional and ethical behavior in mental health settings (p.20, Section 4.5.4) • Nonjudgmental stance toward clients (implied in p.5, Section 1.7) • Respect for client autonomy and dignity (p.5, Section 1.7)
**Sections 4.2 and 4.4: Theoretical Frameworks, Intervention, and Therapeutic Relationships**		
• Knowledge of psychosocial frameworks (p.17, Section 4.2) • Understanding biopsychosocial approaches (p.17, Section 4.2) • Knowledge of direct/indirect service delivery (p.20, Section 4.4)	• Designing interventions for mental health conditions (p.19, Section 4.5.1) • Applying occupation as therapeutic means (p.19, Section 4.5.1) • Implementing interventions to facilitate participation (p.19, Section 4.5.1) • Adapting methods to promote occupational engagement (p.19, Section 4.5.1)	• Empathy and compassion toward psychological distress (p.5, Section 1.7) • Cultural sensitivity when addressing mental health (p.6, Section 1.11) • Self‐awareness of personal reactions (implied in p.5, Section 1.7)
**Sections 4.5.1 and 4.5.2: Person-Occupation-Environment, Therapeutic Use of Self, and Recovery**		
• Understanding how mental health affects occupation (p.19, Section 4.5.1) • Knowledge of environmental influences (p.19, Section 4.5.1) • Understanding social determinants of mental health (p.19, Section 4.5.1)	• Establishing therapeutic relationships (p.19, Section 4.5.2) • Building interpersonal relationships with clients (p.19, Section 4.5.2) • Using effective communication with distressed clients (p.6, Section 1.8)	• Belief in recovery possibilities (implied in p.19, Section 4.5.1) • Hope‐inspiring stance in acute situations (implied in p.19, Section 4.5.1) • Respect for diverse recovery pathways (implied in p.20, Section 4.5.4)
**Section 4.5.4: Clinical Reasoning, Risk, and Cultural Competence**		
	• Interpreting complex mental health situations (p.20, Section 4.5.4) • Applying creativity to solve problems (p.20, Section 4.5.4) • Adapting OT process for mental health conditions (p.20, Section 4.5.4) • Identifying and managing mental health risks (implied in p.15, Section 3.4) • Implementing safety protocols (implied in p.15, Section 3.4) • Documenting and communicating risk (p.11, Section 3.2.8) • Conducting OT group work for mental health (p.20, Section 4.5.1) • Designing group interventions (p.19, Section 4.5.1)	• Commitment to ethical standards (p.5, Section 1.7) • Ability to navigate ethical dilemmas (p.20, Section 4.5.4) • Balancing risk management with autonomy (implied in p.5, Section 1.7) • Sensitivity to diverse cultural perspectives (p.20, Section 4.5.4) • Respect for cultural influences on presentation (p.20, Section 4.5.4) • Openness to various cultural approaches (p.20, Section 4.5.4)

**Table 5:** Competency extraction: WHO Recommendations for Occupational Therapy Practice in Mental Health [39].

Competency area	Knowledge	Skills	Attitudes	Source page(s)
1. Person‐centered and recovery‐oriented care	• Understanding of recovery‐oriented frameworks and principles • Knowledge of personal recovery journeys and stages • Understanding of occupational disruptions related to mental health crises	• Implementation of recovery‐oriented approaches in daily practice • Collaborative goal‐setting with service users • Supporting meaningful identity through occupational engagement	• Commitment to respecting service user autonomy and dignity • Valuing lived experience and personal narratives • Belief in the capacity for recovery and meaningful participation	pp. 42–45, 89–90, 23–24
2. Rights‐based approaches	• Knowledge of human rights frameworks in mental health care • Understanding of legal frameworks for mental health • Knowledge of least restrictive practice principles	• Advocating for least restrictive environments • Supporting informed decision‐making during crises • De‐escalation and noncoercive intervention techniques	• Commitment to human rights protection • Respect for dignity even in restrictive settings • Commitment to minimizing restrictive practices	pp. 32–38, 67–69
3. Comprehensive assessment skills	• Understanding of occupational performance assessment • Knowledge of environment‐occupation relationships • Understanding of risk assessment frameworks	• Functional and occupational assessment in acute settings • Assessment of environmental impacts on functioning • Balancing risk management with promoting independence	• Holistic view of the person beyond symptoms • Recognition of contextual influences on recovery • Positive risk‐taking approach when appropriate	pp. 51–53, 70–72, 78–80
4. Interdisciplinary collaboration	• Knowledge of role boundaries within multidisciplinary teams • Understanding of integrated care models • Understanding of OT′s unique contribution to mental health	• Effective communication of OT perspectives to team members • Navigation of complex healthcare systems • Articulation of occupation‐based approaches to recovery	• Openness to interdisciplinary collaboration • Respect for complementary expertise • Professional advocacy for occupational perspectives	pp. 110–118
5. Evidence‐based psychosocial interventions	• Understanding of evidence‐based psychosocial interventions • Knowledge of occupation‐based intervention models • Understanding of meaningful activity for stabilization	• Adaptation of interventions for acute mental health environments • Integration of occupational perspectives into crisis management • Implementation of graded activity interventions	• Commitment to evidence‐informed practice • Innovation in therapeutic approaches • Belief in occupation as therapeutic medium	pp. 91–99
6. Cultural and contextual competence	• Knowledge of diverse cultural expressions of mental distress • Understanding of social determinants affecting mental health • Knowledge of culturally significant occupations	• Culturally responsive assessment and intervention • Adaptation of interventions to diverse contexts • Cultural safety in therapeutic relationships	• Respect for diversity in occupational choices and values • Recognition of systemic barriers to occupational justice • Cultural humility and ongoing learning	pp. 57–65
7. Trauma‐informed care	• Understanding of trauma′s impact on occupation and functioning • Knowledge of trauma‐informed principles • Understanding of trauma processing through occupation	• Creation of safe therapeutic environments • Trauma‐sensitive assessment approaches • Supporting trauma recovery through meaningful activity	• Trauma‐sensitive approach to all interactions • Commitment to preventing re‐traumatization • Recognition of resilience and healing capacity	pp. 74–81
8. Digital Mental health competencies	• Knowledge of digital mental health tools and resources • Understanding of digital literacy barriers • Knowledge of digital ethical considerations	• Application of telehealth and digital interventions • Adaptation of OT interventions to virtual formats • Ensuring privacy and security in digital practice	• Willingness to innovate and adapt to technological changes • Commitment to digital accessibility • Balanced view of technology benefits and limitations	pp. 102–109
9. Transition and continuity of care	• Understanding of transition pathways from acute to community care • Knowledge of community resources for continued recovery • Understanding of relapse prevention through occupation	• Development of sustainable occupation‐based recovery plans • Evaluation of home and community environments • Facilitation of successful community reintegration	• Commitment to continuity of care across settings • Belief in community integration importance • Long‐term recovery perspective	pp. 120–128
10. Self‐care and professional resilience	• Knowledge of compassion fatigue and professional boundaries • Understanding of vicarious trauma in acute settings • Understanding of ethical considerations in acute mental health	• Implementation of reflective practice and self‐care strategies • Engagement in professional supervision • Ethical decision‐making in complex situations	• Recognition of personal limitations and need for self‐care • Valuing professional wellbeing • Ethical mindset prioritizing service user wellbeing	pp. 132–140

**Table 6:** Competency extraction: WHO mhGAP Intervention Guide Version 2.0 (2016).

Competency area	Knowledge	Skills	Attitudes	Source page(s)
1. Assessment and management of priority conditions	• Understanding of clinical features and diagnostic criteria for priority mental health conditions • Knowledge of functional impact of mental health conditions on daily occupations • Understanding of evidence‐based interventions for priority conditions	• Screening and identification of signs/symptoms of priority mental health conditions • Conducting comprehensive functional assessments for persons with acute mental conditions • Implementation of targeted psychosocial interventions for specific conditions	• Commitment to evidence‐based assessment approaches • Recognition of the person beyond their diagnosis • Belief in recovery potential for all service users	pp. 18–20, 23–27, 30–32
2. General principles of care	• Knowledge of effective communication strategies in mental health contexts • Understanding of person‐centered assessment frameworks • Knowledge of community resources and support systems • Understanding of family dynamics in mental health care	• Communication with people experiencing mental health crises • Conducting assessments in a recovery‐oriented manner • Mobilizing community resources to enhance recovery • Involving family members and caregivers appropriately in care	• Respect for dignity and autonomy • Empathy and nonjudgmental approach to care • Belief in community integration as essential to recovery • Recognition of family as partners in care	pp. 10–17, 40–45
3. Advanced psychosocial interventions	• Knowledge of behavioral activation principles and techniques • Understanding of stress management and relaxation techniques • Knowledge of cognitive‐behavioral principles relevant to occupation • Understanding of family systems and psychoeducation approaches	• Implementation of behavioral activation for depression • Application of stress management interventions • Integration of cognitive‐behavioral techniques in occupational interventions • Facilitation of family psychoeducation and support	• Commitment to active, participatory approaches • Belief in self‐management capacity • Value of cognitive approaches in behavioral change • Recognition of family as a recovery resource	pp. 86–102
4. Crisis management	• Knowledge of de‐escalation principles and techniques • Understanding of suicide risk factors and safety protocols • Knowledge of acute psychosis management principles • Understanding of risk assessment frameworks in acute mental health	• Application of de‐escalation techniques for agitated individuals • Implementation of safety measures for suicidal individuals • Responding to acute psychosis using least restrictive approaches • Assessment and management of risk in acute settings	• Calm and nonreactive approach to crisis • Belief in prevention potential • Commitment to human rights in crisis situations • Balanced approach to risk and autonomy	pp. 50–52, 55–58, 62–70
5. Recovery and social support	• Knowledge of social determinants of mental health • Understanding of life skills development frameworks • Knowledge of environmental modification principles • Understanding of occupational justice principles	• Facilitation of access to education, employment, and social activities • Supporting independent living and developing essential life skills • Adaptation of environments to support functioning • Addressing social barriers affecting mental health recovery	• Belief in social inclusion as central to recovery • Value of independence and self‐determination • Recognition of environment′s role in disability • Commitment to occupational rights	pp. 110–126
6. Integration into health systems	• Knowledge of mental health service structures and pathways • Understanding of interdisciplinary team roles and functions • Knowledge of advocacy principles and approaches • Understanding of effective clinical documentation	• Navigation of referral pathways and appropriate specialist engagement • Collaboration within multidisciplinary mental health teams • Advocacy for mental health service users within healthcare systems • Documentation and communication with the healthcare team	• Commitment to coordinated care • Value of diverse professional perspectives • Belief in equal access to quality care • Respect for information sharing ethics	pp. 130–147
7. Depression interventions	• Knowledge of activity scheduling principles for depression • Understanding of psychosocial stressors and their functional impact • Knowledge of social engagement strategies for depression	• Implementation of activity scheduling and graded task assignments • Identification and addressing of stressors affecting functioning • Promotion of social engagement and meaningful occupation	• Belief in activity as therapeutic for mood • Recognition of social context in depression • Value of social connection in recovery	pp. 160–172
8. Psychosis interventions	• Understanding of rehabilitation approaches for psychosis • Knowledge of cognitive remediation principles • Understanding of sensory modulation in psychosis	• Implementation of activity‐based rehabilitation interventions • Addressing cognitive deficits through structured activities • Environmental modifications to reduce overstimulation	• Belief in functional recovery in psychosis • Recognition of cognitive capacity despite illness • Sensitivity to environmental impacts	pp. 180–192
9. Substance use interventions	• Knowledge of motivational interviewing principles • Understanding of routine development in addiction recovery • Knowledge of occupational balance principles	• Application of motivational interviewing techniques • Development of structured routines that reduce triggers • Addressing occupational imbalance in recovery	• Nonjudgmental approach to substance use • Belief in structured occupation as protective • Recognition of meaningful activity as central to recovery	pp. 200–212
10. Self‐harm/suicide interventions	• Understanding of environmental safety assessment principles • Knowledge of meaningful activity planning for suicide prevention • Understanding of collaborative safety planning principles	• Assessment of home environment safety • Development of meaningful activity plans that promote hope • Implementation of safety planning with clients and families	• Proactive approach to safety planning • Belief in purpose as protective factor • Partnership approach to risk management	pp. 220–232
11. Dementia interventions	• Knowledge of cognitive stimulation therapy principles • Understanding of environmental design for dementia • Knowledge of caregiver training approaches	• Application of cognitive stimulation therapies • Environmental modification for safety and orientation • Training caregivers in managing behavioral symptoms	• Belief in cognitive reserve and plasticity • Recognition of environment as therapeutic tool • Respect for caregiver expertise and experience	pp. 240–252

#### 2.4.2. Stage 2: UKZN Exit‐Level Outcome Extraction

The UKZN exit‐level outcomes were extracted using the same KSA framework applied in Stage 1, with competency domains mapped to match the categories identified across the guiding frameworks. The UKZN extraction is presented in Table 7. It is important to note that the UKZN curriculum data source used in this study was exclusively the program′s official exit‐level outcomes document (2024 version), obtained with institutional permission. Exit‐level outcomes were selected as the data source because they represent the formal, authoritative statement of what graduates are expected to know, do, and be upon completion of the degree—they are the regulatory interface between what is taught and what is accredited. Although acknowledging that module guides, lecture content, and fieldwork experiences may address some competency content not captured at the exit‐level outcomes level, the outcomes document is the legitimate comparator for regulatory and accreditation purposes. Alignment was determined by whether a given competency domain was explicitly named, described, or operationalized within the exit‐level outcomes statements; implicit or inferential presence was coded as “partial alignment” rather than “strong alignment.” Complete absence from any outcome statement was coded as a “gap.” These operational definitions were documented in the analytical audit trail and reviewed by both supervisors.

**Table 7:** Competency extraction: UKZN undergraduate occupational therapy program exit level outcomes.

Competency area	Knowledge	Skills	Attitudes
Theoretical and philosophical base	• Understanding of occupational therapy theories relevant to mental health • Knowledge of direct and indirect service approaches • Understanding of South African mental health context	• Application of theories to address occupational needs in mental health • Translation of theoretical principles into practical interventions	• Valuing theoretical foundations of practice • Recognition of the importance of occupational needs in mental health recovery
Clinical reasoning and intervention	• Knowledge of therapeutic media and techniques for mental health • Understanding of psychosocial approaches • Knowledge of group dynamics and processes	• Adaptation of OT process using professional reasoning • Implementation of appropriate therapeutic media and techniques • Facilitation of therapeutic groups • Delivery of services across age groups • Establishment of services in psychosocial fields	• Critical thinking approach to intervention • Flexibility in adapting techniques • Commitment to evidence‐informed intervention
Cultural competence in assessment	• Understanding of cultural influences on mental health • Knowledge of assessment approaches for diverse populations • Understanding of planning and implementation rationales	• Adaptation of interventions for specific cultural needs • Assessment and analysis skills • Planning and implementation for different levels of care • Intervention for large numbers and diverse needs	• Cultural humility • Respect for diversity • Openness to adapting practice based on cultural context
Autonomous practice in complex contexts	• Understanding of diverse practice contexts in mental health • Knowledge of complexities in mental health settings	• Working effectively in unfamiliar contexts • Autonomous decision‐making • Communication in challenging environments	• Professional identity • Confidence in own abilities • Adaptability to complex situations
Interdisciplinary collaboration	• Knowledge of team functioning in mental health settings • Understanding of roles of different stakeholders	• Effective communication with team members • Collaboration with relevant stakeholders • Integration of OT perspective in team settings	• Respect for other disciplines • Value of team‐based care • Openness to different perspectives
Ethical practice	• Knowledge of ethical principles for mental health practice • Understanding of legislative frameworks • Knowledge of HPCSA requirements	• Ethical decision‐making • Operating within professional guidelines • Taking responsibility for actions	• Commitment to ethical standards • Professional integrity • Accountability
Reflective practice	• Understanding of reflection principles • Knowledge of self‐improvement methods	• Critical reflection on own practice • Adaptation of approach based on reflection • Self‐evaluation skills	• Openness to feedback • Commitment to ongoing improvement • Self‐awareness
Advocacy	• Knowledge of health and social justice issues in mental health • Understanding of advocacy principles	• Advocating for/with clients • Collaborative advocacy skills • Communication for advocacy purposes	• Commitment to social justice • Client‐centered values • Drive to address systemic issues
Person‐occupation‐environment relationship	• Understanding of P‐O‐E framework in mental health • Knowledge of OT principles in South African context • Awareness of international trends in mental health OT	• Application of P‐O‐E concepts in practice • Critical evaluation of principles and policies • Adaptation of practice to South African context	• Holistic perspective • Recognition of environmental influences • Systems thinking approach
Evidence‐based practice	• Knowledge of relevant literature and sources • Understanding of evidence hierarchy • Knowledge of best practices in mental health OT	• Critical review of literature • Application of evidence to practice • Integration of research into clinical reasoning	• Commitment to evidence‐based practice • Scientific mindset • Intellectual curiosity
Health and educational trends	• Knowledge of health, welfare, and educational policies • Understanding of lifespan development in mental health • Awareness of service provision trends	• Application of policies across lifespan • Analysis of trends′ impact on OT services • Adaptation of practice to policy contexts	• Forward‐thinking approach • Recognition of policy influences • Commitment to relevant service provision

#### 2.4.3. Stage 3: Cross‐Framework Synthesis of Common Competency Areas

Following individual extraction, the four guiding documents (3–6) were examined together to identify key competency areas that appeared consistently or prominently across frameworks. Seven core competency domains emerged from this synthesis: (1) recovery‐oriented and person‐centered practice; (2) assessment skills; (3) therapeutic use of self and communication; (4) knowledge of mental health conditions and interventions; (5) risk assessment and management; (6) cultural competence; and (7) interdisciplinary collaboration. For each domain, competencies from all four frameworks were arranged side by side to reveal patterns of convergence and divergence in how the domain was conceptualized, detailed, and weighted. This synthesis is presented in Table 8 in the findings section.

**Table 8:** Summary of common competency domains across guiding frameworks.

Competency domain	WFOT	HPCSA	WHO Rec.	WHO mhGAP
Recovery‐oriented and person‐centered practice	✔ Explicit—distinct competency area (pp. 27, 42)	✔ Implied—embedded in therapeutic relationships (p. 19, s4.5.2)	✔ Explicit—first and primary domain (pp. 42–45, 89–90)	✔ Explicit—embedded in general principles (pp. 15–17, 40–45)
Assessment skills	✔ Explicit—distinct competency area (pp. 22–23, 38)	✔ Explicit—holistic and functional assessment (p. 19, s4.5.1)	✔ Explicit—occupational performance and environmental assessment (pp. 51–53, 78–80)	✔ Explicit—screening, functional and home environment assessment (pp. 18–27, 220–222)
Therapeutic use of self and communication	✔ Explicit—distinct competency area (pp. 17–18, 32)	✔ Explicit—therapeutic relationships (p. 19, s4.5.2; p. 6, s1.8)	✔ Distributed—within cultural competence and trauma‐informed care (pp. 63–65, 74–76)	✔ Embedded—within general principles of care (pp. 10–14)
Knowledge of mental health conditions and interventions	✔ Explicit—psychiatric conditions, psychopharmacology (pp. 28–29, 43)	✔ Explicit—DSM/ICD knowledge, etiology, management (pp. 14–15, s3.4)	✔ Explicit—evidence‐based psychosocial interventions (pp. 91–99)	✔ Highly detailed—condition‐specific chapters (pp. 160–252)
Risk assessment and management	✔ Explicit—distinct competency area including suicide, violence, safety (pp. 30, 44–45)	✔ Implied—within psychiatric conditions section (p. 15, s3.4)	✔ Explicit—risk‐benefit frameworks, least restrictive practice (pp. 67–72)	✔ Explicit—safety planning, de‐escalation, environmental safety (pp. 50–70, 220–232)
Cultural competence	✔ Explicit—distinct competency area (pp. 16, 37)	✔ Attitudinal only ‐sensitivity and respect (p. 6, s1.11; p. 20, s4.5.4)	✔ Explicit—culturally significant occupations, responsive practice (pp. 57–65)	✔ Distributed—integrated across condition‐specific domains
Interdisciplinary collaboration	✔ Explicit—distinct competency area (pp. 35–36, 48)	✗ Not explicitly detailed as a separate competency	✔ Explicit—role boundaries, integrated care models (pp. 110–118)	✔ Explicit—referral pathways, team collaboration (pp. 130–137)

#### 2.4.4. Stage 4: Gap Analysis

The final stage involved a systematic gap analysis in which competency domains consistently present across international frameworks but absent or insufficiently addressed in the UKZN outcomes were identified and documented. Gaps were defined as competency areas (a) explicitly articulated in two or more of the four guiding frameworks, and (b) absent from, or only implicitly addressed within, the UKZN exit‐level outcomes. Six such gaps were identified, each with direct implications for curriculum enhancement and are presented in the findings section (Table 8). Alignment decisions were guided by three predefined criteria operationalized prior to beginning the mapping process and documented in the analytical audit trail. “Strong alignment” was assigned when a UKZN exit‐level outcome explicitly named, described, or operationalized the competency domain in terms of specific knowledge, skills, or attitudes with comparable scope and depth to the guiding frameworks. “Partial alignment” was assigned when the competency domain was present in the UKZN outcomes at a general or implicit level, but lacked the acute mental health‐specific articulation present in the guiding frameworks (for example, professional communication skills addressed generically rather than as de‐escalation or crisis communication specifically). “Gap” was assigned when the competency domain was entirely absent from the exit‐level outcomes or was so peripherally referenced that no reasonable interpretation of the outcome could be understood to address it. These decisions were not made by the first author alone; all alignment judgments were reviewed by both supervisors as part of the peer debriefing process described above, providing an external verification mechanism for the mapping outcomes.

### 2.5. Ethical considerations and Methodological Rigor

The first author occupies a dual position as both a doctoral researcher and a practicing occupational therapist at an acute psychiatric facility, with familiarity with the UKZN program′s educational context. This insider positioning brings methodological advantages including deep contextual knowledge, but also requires explicit management of potential bias, particularly given that the study evaluates a framework belonging to the researcher′s own institution.

Therefore, procedural safeguards were implemented to strengthen the confirmability and credibility of the analysis. First, a reflexive journal was maintained throughout the analytical process, in which the first author documented assumptions, interpretive decisions, and potential biases at each stage. Second, all analytical decisions, including competency extraction categories, inclusion and exclusion criteria, and alignment judgments, were documented in a comprehensive audit trail to ensure transparency and replicability. Third, the analysis benefited from ongoing peer debriefing with two study supervisors (coauthors), who reviewed the competency extraction tables, challenged interpretive conclusions, and provided independent perspectives on the alignment and gap findings throughout the process. This supervisory review served as a form of external scrutiny of the analytical outputs, particularly important given the institutional stake in the UKZN framework. Fourth, the structured, multistage analytical procedure itself, in which extraction, synthesis, mapping, and gap identification were conducted as sequential, documented steps rather than as a single interpretive exercise, provided a procedural basis for confirmability independent of individual researcher judgemnt. Ethical approval for this study was granted by the UKZN Biomedical Research Ethics Committee (Reference: BREC/00004840/2022). All documents analyzed were either publicly available or accessed with appropriate institutional permissions.

## 3. Findings

The findings are presented in three parts, following the analytical procedure described above. Part 1 presents the structural characteristics of each framework. Part 2 reports the common competency domains identified across the guiding frameworks. Part 3 presents the alignment of UKZN exit‐level outcomes with these domains and identifies and details the gaps in the UKZN framework relative to international standards.

### 3.1. Part 1: Structural Approaches to Competency Organization Across Frameworks

All five frameworks organized competencies using the knowledge–skills–attitudes (KSA) structure, though with notably different emphases. The WFOT framework provided the most balanced coverage across all three KSA domains, with comparable depth of knowledge, skills, and attitudes articulated for each of its 10 competency areas. This balance reflects WFOT′s aim to produce a globally applicable standard that attends equally to what practitioners *know*, what they can *do*, and how they *approach* their work. The HPCSA framework placed proportionally greater emphasis on knowledge competencies, with more limited articulation of corresponding skills and attitudes. Attitudes, in particular, were largely implied rather than explicitly stated, appearing most often in the document′s general principles section rather than within specific competency domains. This pattern is consistent with a regulatory document whose primary function is to define minimum knowledge thresholds for professional registration rather than to describe the full professional character of a competent practitioner. The WHO Recommendations framework demonstrated high balance across KSA domains, with particular strength in connecting attitudinal competencies to specific practice contexts. For example, articulating not merely that practitioners should demonstrate cultural humility, but specifying what that humility look like in acute assessment contexts. The WHO mhGAP provided the most detailed condition‐specific knowledge and practical skills, organized by clinical presentation (depression, psychosis, substance use, self‐harm, and dementia) rather than by professional competency domain. This reflects the mhGAP′s function as an implementation guide for frontline practitioners in LMICs rather than as a professional education framework. The UKZN exit‐level outcomes integrated competencies within a broader educational framework structured around professional roles and graduate attributes, with notable emphasis on reflective practice, advocacy, and person‐occupation‐environment reasoning as distinct competency areas. Unlike the guiding frameworks, UKZN′s outcomes were not organized explicitly by KSA domain within each competency area, though KSA elements were present across the outcomes when mapped using the extraction framework.

### 3.2. Part 2: Common Competency Domains Across Guiding Frameworks

Cross‐framework synthesis of the four guiding documents identified seven core competency domains present across all or most frameworks. The comparative analysis revealed that no single framework provided a complete or sufficient account of all seven domains. Rather, the frameworks were found to be complementary, each contributing distinct emphases that, in combination, produced a more complete picture of required competence than any document offered in isolation. This cross‐framework synthesis was therefore not simply confirmatory but generative: placing the documents in dialogue with one another surfaced patterns of convergence and divergence that would not have been apparent from reading each framework independently.

Several insights emerged from this process. First, recovery‐oriented and person‐centered practice was affirmed across all four frameworks, yet the nature of that affirmation varied significantly. The WHO recommendations provided the most operationalized articulation of recovery principles, with detailed guidance on collaborative goal setting and the meaningful integration of lived experience, whereas WFOT distinguished itself by explicitly linking recovery to occupational engagement, a connection absent in the WHO documents. This divergence revealed that “recovery‐orientation” is not a uniform construct across frameworks but is shaped by each document′s underlying mission and conceptual orientation. For occupational therapy education specifically, this finding highlights the importance of drawing from multiple frameworks rather than defaulting to a single authoritative source.

Second, the analysis revealed a consistent and notable gap in how the HPCSA framework addressed certain domains. Interdisciplinary collaboration, treated as an explicit, distinct competency by WFOT and both WHO frameworks, was not separately delineated in the HPCSA document. Similarly, cultural competence in the HPCSA framework was limited to attitudinal descriptors such as sensitivity and respect, with no corresponding knowledge or skills content. This pattern suggested that the HPCSA framework, whereas essential as a regulatory reference for South African practice, is insufficient as a standalone guide for acute mental health competency development and requires supplementation with international and global health perspectives.

Third, the synthesis illuminated a structural distinction between frameworks that treat certain competencies as discrete domains and those that embed the same content across other areas. Therapeutic use of self and communication, for instance, was positioned as an explicit standalone domain in WFOT and HPCSA, but was distributed across cultural competence, trauma‐informed care, and person‐centered practice sections in the WHO documents. This finding carries implications for curriculum design: integrated competency embedding may support holistic practice but risks reduced visibility and under‐assessment of foundational skills unless explicitly mapped.

Taken together, the cross‐framework analysis demonstrated that meaningful preparation for acute mental health practice cannot be grounded in any single guiding document. The convergence across frameworks on the shared domains affirms the core knowledge, skills, and attitudes that graduates must develop. The divergences in emphasis, explicitness, and structural placement reveal where individual frameworks are incomplete, and where the integration of multiple perspectives is necessary to produce competency standards adequate to the demands of acute mental health practice in the South African context.

Table 8 below summarizes the key patterns of convergence and divergence.

### 3.3. Part 3: Alignment of UKZN Exit‐Level Outcomes With Guiding Frameworks

Table 9 below presents the alignment of UKZN exit‐level outcome areas against the seven common competency domains identified in Part 2. Three categories of alignment are distinguished: strong alignment (the UKZN outcome explicitly and substantively addresses the domain); partial alignment (the UKZN outcome addresses elements of the domain but with less specificity or only implicitly); and gap (the domain is absent from, or insufficiently represented in, the UKZN outcomes relative to the guiding frameworks).

**Table 9:** Alignment of UKZN exit‐level outcomes with competency domains identified in guiding frameworks.

#	Competency domain	Guiding framework coverage	UKZN alignment	Finding
**Strong alignment (two domains)**				
**1**	**Cultural competence**	**WFOT:** ✔ full **HPCSA:** ◑ partial **WHO Rec.:** ✔ full **WHO mhGAP:** ◑ partial	**✔ STRONG** Explicitly and substantively addressed as a discrete competency.	Cultural competence in assessment is a dedicated UKZN competency area with explicit knowledge, skills, and attitudes addressing diverse cultural contexts, assessment adaptation across cultural settings, and cultural humility in therapeutic relationships. This represents the most robust alignment between the UKZN framework and the guiding documents.
**2**	**Interdisciplinary collaboration**	**WFOT:** ✔ full **HPCSA:** absent **WHO Rec.:** ✔ full **WHO mhGAP:** ✔ full	**✔ STRONG** Explicitly addressed as a distinct competency area.	Interdisciplinary Collaboration is a named UKZN competency area specifying team communication, stakeholder collaboration, and integration of the occupational therapy perspective in multidisciplinary team settings. Despite being absent from HPCSA guidelines, UKZN coverage aligns with WFOT, WHO recommendations, and WHO mhGAP.
**Partial alignment (four domains)**				
**3**	**Recovery-oriented and person-centered practice**	**WFOT:** ✔ full **HPCSA:** ◑ partial **WHO Rec.:** ✔ full **WHO mhGAP:** ✔ full	**⚠ PARTIAL** Present implicitly; not articulated as a distinct domain.	Recovery principles are embedded within the P‐O‐E relationship and clinical reasoning competencies but are not articulated as a distinct domain. No UKZN outcome specifies skills for supporting hope, preserving client autonomy within restricted inpatient environments, or reconnecting individuals with valued occupational roles during acute admission, competencies explicitly required by WFOT and WHO recommendations.
**4**	**Assessment skills**	**WFOT:** ✔ full **HPCSA:** ✔ full **WHO Rec.:** ✔ full **WHO mhGAP:** ✔ full	**⚠ PARTIAL** Cultural assessment addressed; acute‐specific assessment absent.	Cultural competence in assessment is addressed as a discrete UKZN competency, demonstrating strong alignment on this dimension. However, no outcome specifies standardized psychiatric assessment instruments, occupational performance evaluations for psychiatric populations, or time‐limited assessment approaches suited to acute inpatient settings, all required by WFOT, HPCSA, and WHO mhGAP.
**5**	**Therapeutic use of self and communication**	**WFOT:** ✔ full **HPCSA:** ✔ full **WHO Rec.:** ◑ partial **WHO mhGAP:** ◑ partial	**⚠ PARTIAL** General therapeutic communication present; crisis‐specific skills absent.	The therapeutic relationship and professional communication are implicitly embedded within clinical reasoning and autonomous practice. However, specific communication competencies required in acute settings: de‐escalation techniques, crisis communication, and maintaining therapeutic presence during acute psychological distress, are not specified in any UKZN outcome, despite being required by WFOT, WHO recommendations, and WHO mhGAP.
**6**	**Professional resilience and self-care**	**WFOT:** absent **HPCSA:** absent **WHO Rec.:** ✔ Full **WHO mhGAP:** absent	**⚠ PARTIAL** General reflective practice present; acute‐specific self‐care absent.	The reflective practice competency addresses self‐evaluation, professional development, and adaptation. However, it does not address the specific emotional demands of acute mental health work. Competencies related to recognizing compassion fatigue, managing vicarious trauma, and implementing self‐care strategies in high‐stress psychiatric settings are absent, despite being explicitly included by WHO recommendations.
**Confirmed gaps (six domains)—absent from UKZN exit-level outcomes**				
**7**	**Knowledge of acute mental health conditions and condition-specific interventions**	**WFOT:** ✔ full **HPCSA:** ✔ full **WHO Rec.:** ✔ full **WHO mhGAP:** ✔ full	**✗ GAP** Entirely absent from UKZN outcomes.	This is the most extensively evidenced gap, required in full by all four guiding frameworks. WFOT requires knowledge of acute psychiatric conditions and psychopharmacology effects on occupational performance HPCSA requires DSM/ICD knowledge, aetiology, clinical presentation, and psychopharmacology. WHO mhGAP specifies condition‐specific chapters for depression, psychosis, substance use, and dementia including behavioral activation, graded task assignment, and activity‐based rehabilitation. The UKZN theoretical and philosophical Base competency references OT theories and South African mental health context but does not specify acute diagnoses, symptom presentation, psychopharmacological effects on functioning, or condition‐specific interventions. This gap is foundational, without it, other acute competencies cannot be meaningfully applied. Its absence means graduates enter acute settings without the foundational clinical knowledge needed to connect psychiatric presentations to occupational functioning or to select and adapt condition‐specific intervention approaches.
**8**	**Risk assessment and safety management**	**WFOT:** ✔ full **HPCSA:** ◑ partial **WHO Rec.:** ✔ full **WHO mhGAP:** ✔ full	**✗ GAP** Entirely absent from UKZN outcomes.	Required by three guiding frameworks, this competency is absent from every UKZN exit‐level outcome. WFOT requires suicide risk factors, violence risk assessment, safety protocols, de‐escalation skills, and risk documentation. WHO mhGAP specifies safety measures for suicidal individuals, de‐escalation for agitated individuals, safety planning, and home environment assessment. WHO recommendations require least restrictive practice and risk‐benefit frameworks. In acute mental health settings, occupational therapists routinely encounter individuals presenting with suicide risk, self‐harm, or potential for violence. The absence of this competency is not only a curriculum gap, it is a patient safety concern that warrants priority attention in any revision process. The autonomous practice in complex contexts competency references challenging environments but does not specify safety management or risk‐related decision‐making.
**9**	**Trauma-informed care**	**WFOT:** ◑ partial **HPCSA:** absent **WHO Rec.:** ✔ full **WHO mhGAP:** ◑ partial	**✗ GAP** Entirely absent from UKZN outcomes.	WHO recommendations require trauma′s impact on occupation and functioning, creating safe therapeutic environments, trauma‐sensitive assessment, preventing retraumatization, and supporting trauma recovery through meaningful activity. WFOT lists trauma‐informed approaches within the therapeutic use of self‐competency. Trauma‐informed care does not appear as a competency category or as a specified knowledge or skill element in any UKZN exit‐level outcome. Many acute admissions are themselves traumatic experiences, and graduates without training in trauma‐sensitive assessment and the prevention of retraumatization risk causing inadvertent harm through standard approaches that have not been adapted for trauma.
**10**	**Crisis intervention and de-escalation**	**WFOT:** ✔ full **HPCSA:** absent **WHO Rec.:** ◑ partial **WHO mhGAP:** ✔ full	**✗ GAP** Entirely absent from UKZN outcomes.	WFOT specifies de‐escalation techniques and crisis intervention as distinct required skills. WHO mhGAP requires application of de‐escalation for agitated individuals and least restrictive responses to acute psychosis. WHO Recommendations identify de‐escalation and noncoercive intervention as core competencies. These represent day‐to‐day competencies in acute settings, yet they do not appear in any UKZN outcome. The current Autonomous Practice competency references challenging environments but does not specify the skills required to navigate them safely. There is a meaningful clinical difference between tolerating a difficult environment and being able to de‐escalate an agitated patient or respond to acute psychosis using least restrictive approaches.
**11**	**Digital mental health competencies**	**WFOT:** absent **HPCSA:** absent **WHO Rec.:** ✔ full **WHO mhGAP:** absent	**✗ GAP** Entirely absent from UKZN outcomes.	WHO Recommendations address digital mental health tools and resources, telehealth application, digital intervention adaptation, digital accessibility, and digital privacy and security in practice. This domain is not yet addressed in WFOT, HPCSA, or WHO mhGAP, making WHO recommendations the primary reference. Nevertheless, digital mental health is entirely absent from the UKZN exit‐level outcomes. Although this gap is less clinically urgent than the preceding five, telehealth and digital intervention platforms are already present in South African mental health services, and it represents a forward‐facing gap that requires proactive curriculum planning.
**12**	**Transition and continuity of Care planning**	**WFOT:** absent **HPCSA:** Absent **WHO Rec.:** ✔ full **WHO mhGAP:** ◑ partial	**✗ GAP** Entirely absent from UKZN outcomes.	WHO recommendations require developing occupation‐based recovery plans for acute‐to‐community transitions, evaluating home and community environments, facilitating community reintegration, and knowledge of community resources for recovery. WHO mhGAP addresses community follow‐up and continuity of care for priority conditions. This gap is absent despite being the structural purpose of occupational therapy in acute settings. Acute admission is time‐limited; the OT role is to stabilize, assess, and plan for a return to community functioning. Discharge planning, home environment evaluation, and community reintegration facilitation are not incidental skills, they define what acute OT is for. Their absence suggests the transitional nature of acute practice has not been made explicit in the outcomes framework.

## 4. Discussion

The findings of this study carry two analytically distinct kinds of significance. The first concerns what the comparative synthesis reveals about the occupational therapy frameworks: no single document currently offers a complete account of the competencies required for acute mental health occupational therapy practice. The HPCSA framework, the primary regulatory reference for all eight South African occupational therapy programs, does not separately delineate interdisciplinary collaboration as a competency, addresses cultural competence at the attitudinal level only, and implies rather than specifies risk assessment, in contrast to WFOT [38] and WHO mhGAP [40] which treat it as a nonnegotiable standalone domain. Mavindidze et al. [32], Mabuza [31], and Crouch and Alers [30] have identified a tendency toward generic mental health competency specification in South African regulatory standards. The study findings establish that this tendency originates in the regulatory framework itself, not only in how programs have interpreted it. Furthermore, where all four frameworks agree on a domain′s importance, they frequently disagree on what it requires in practice. Recovery‐oriented practice is affirmed across all four, yet each operationalizes it differently: WFOT [38] links recovery to occupational engagement; WHO mhGAP [41] embeds it within condition‐specific management for LMIC frontline contexts. These divergences confirm that adequate preparation for acute mental health practice cannot be achieved through alignment with any single source, and that the present study produces the comparative basis on which deliberate cross‐framework synthesis can be undertaken in South Africa.

The second level of significance concerns what the UKZN alignment profile reveals about how the program has conceptualized professional preparation. The profile is internally coherent: It is not a random distribution of strengths and gaps but reflects an identifiable model. The two strongly aligned domains, cultural competence and interdisciplinary collaboration, are both domains of professional orientation, and in both, the UKZN outcomes exceed the HPCSA standard (Table 9). This reflects the sustained investment South African occupational therapy education has made in contextually responsive, equity‐oriented practice [18, 19], a genuine strength confirmed empirically by this study′s findings. The four partially aligned domains: recovery‐oriented practice, assessment skills, therapeutic use of self and communication, and professional resilience, share a common finding: the UKZN framework holds the relevant professional value but has not operationalized it into the acute‐specific clinical competency the guiding frameworks require. The P‐O‐E relationship and clinical reasoning outcomes embed recovery principles, but no UKZN outcome specifies what recovery demands of the therapist working with an involuntary patient in a restricted inpatient environment, the critical translation gap between recovery philosophy and recovery‐oriented acute practice that Lloyd and Williams [12] identify. The reflective practice outcome addresses self‐evaluation but omits the specific demands of acute mental health work, recognizing compassion fatigue, managing vicarious trauma, identified by Scanlan and Still [42] as central to sustainable practice in high‐stress settings. This pattern points to a structural orientation in the curriculum: that clinical specificity will follow through practice exposure. Research however, has demonstrated that this approach reliably produces inconsistent graduate readiness; identified it as a systemic tendency in OT frameworks [32, 43]. This study now locates it precisely within the UKZN outcomes structure with multiframework empirical evidence.

The six confirmed gaps intensify this concern. The absence of condition‐specific knowledge (Table 9, Domain 7) is meaningfully prioritized to all other gaps. It is the only domain for which all four guiding frameworks demonstrated full coverage, and without it, other acute competencies cannot be meaningfully deployed. A graduate cannot apply graded task assignment for treatment‐resistant depression or plan community reintegration for a person with acute psychosis without knowing how those conditions present occupationally [12]. What this reveals is that condition‐specific knowledge has been treated as something graduates acquire through clinical experience rather than something the curriculum establishes, a position not teachable when acute psychiatric placement averages 5–7 weeks across 4 years, when specialized mental health teaching is already constrained by faculty expertise and curriculum demands, which supports what literature have documented that newly qualified occupational therapists consistently report unpreparedness for acute psychiatric settings [22, 23, 26]. Risk assessment (Table 9, Domain 8) is absent from all UKZN outcomes despite being required by three of four guiding frameworks, revealing that the curriculum has treated the acute setting as complex in a general professional sense rather than acknowledging it as a qualitatively distinct risk environment, a patient safety concern in settings where encounters with suicidality, self‐harm, and behavioral agitation are routine [7, 44]. The remaining gaps, trauma‐informed care, crisis intervention, and transition planning, together reveal that the curriculum has not yet engaged with what is specifically distinctive about the acute psychiatric setting as a therapeutic environment. Holman et al. [45] document that occupational therapists without trauma‐sensitive training risk inadvertently replicating dynamics of powerlessness with this population, particularly given South Africa′s elevated burden of interpersonal violence and intergenerational trauma. Additionally, crisis management is absent despite Scanlan et al. [24] identifying it as the acute mental health competency where OT graduates internationally report the lowest confidence. Transition planning; what Lloyd et al. [12] and Brown et al. [46] describe as the defining purpose of acute occupational therapy, does not appear in any UKZN outcome, suggesting that acute mental health practice has been treated as a variant of mental health practice in general rather than a distinct context with its own clinical purpose and requirements.

Taken together, these findings describe not a poorly designed curriculum but one built around a coherent model of professional formation that has not yet been extended to include the clinical specificity acute mental health practice requires. The strengths are the foundations of a graduate equipped for the social and professional complexity of South African healthcare [18, 19]. What is absent are the domains that require the graduate to act clinically in an acute context: to assess specific presentations, manage safety, respond to crisis, and plan transitions. The curriculum has operated on the assumption that professional values, once formed, translate into contextually appropriate clinical action through experience. Tables 8 and 9 demonstrate that this assumption does not hold for acute mental health practice, where clinical demands are too specific, placement exposure too brief and the consequences of unpreparedness too serious for that translation to occur without explicit curriculum support [7, 31, 44, 47]. This study′s contribution is not simply to document that gaps exist as the existing literature had signaled most of them, but to establish through systematic multiframework comparison precisely what those competencies require, where their specifications can be found, and why the gap cannot be closed by aligning with any single regulatory document. Addressing it requires drawing on WFOT [38] and WHO Intervention guidelines [40] for the operationalized guidance on partially aligned domains, and on all four frameworks for confirmed gap content. It also requires advocating for the HPCSA accreditation framework to be strengthened, since no program can achieve adequate acute mental health competency preparation through regulatory compliance alone. Pedagogically, simulation‐based learning and structured problem‐based approaches using acute case presentations offer practical routes to developing this competency [27, 28].

## 5. Implications for Practice

The implications of these findings extend beyond the curriculum and carry direct relevance for occupational therapy practice in acute mental health settings across South Africa. Clinicians and supervisors in acute psychiatric facilities currently receive newly qualified occupational therapists whose foundational training has not systematically addressed risk assessment, crisis intervention, trauma‐informed care, or transition planning, placing a disproportionate burden on workplace‐based supervision to develop competencies that should have been established prior to registration. Professional bodies including the HPCSA and OTASA therefore have a responsibility to ensure that minimum competency standards for acute mental health practice are explicitly reflected in educational accreditation requirements and continuing professional development frameworks. At the service delivery level, this preparation gap contributes directly to the mental health occupational therapy workforce shortage as graduates are more likely to gravitate toward practice areas where they feel more confident. Addressing the identified gaps is therefore a workforce retention and service delivery imperative, not only at UKZN but also across all eight South African occupational therapy programs.

## 6. Limitations

Several limitations of this study warrant acknowledgement. The analysis was conducted at a single institution, and findings may not be directly transferable to other programs without independent analysis of their respective outcomes frameworks. As a methodology, document analysis examines what frameworks specify rather than what is actually taught; some identified gaps may be addressed within module content or fieldwork not captured in the exit‐level outcomes document, though the outcomes framework remains the formal statement of graduate competency and its silences carry regulatory significance. The purposive document selection, although systematically applied, excluded condition‐specific South African clinical guidelines, which may mean some competency dimensions were not fully captured. Finally, the first author′s dual role as researcher and practicing occupational therapist at an acute psychiatric facility introduces the possibility of interpretive bias, managed through reflexive journaling, an audit trail, and supervisory peer debriefing throughout the analytical process.

## 7. Conclusion

This study provides the first systematic, multiframework competency mapping of a South African occupational therapy program against national regulatory standards and current international best practice guidance for acute mental health practice. The findings confirm that the UKZN exit‐level outcomes framework embodies genuine professional strengths in cultural competence, interdisciplinary collaboration, ethical practice, and contextual responsiveness. However, the partial alignments and confirmed gaps collectively demonstrate that the framework does not yet provide the acute mental health‐specific competency foundation graduates require to practice safely and effectively in inpatient psychiatric settings from the outset of their careers. Curriculum enhancement should focus on translating existing professional values into explicit, operationalized competency statements that are both globally informed and locally authentic. The most important implication of this study is twofold. First, at the UKZN program level, and by extension all South African occupational therapy programs must urgently revise exit‐level outcomes. These outcomes are to include explicit, acute mental health‐specific competency statements covering condition‐specific knowledge, risk assessment and safety management, trauma‐informed care, crisis intervention and de‐escalation, and discharge and transition planning. These are not peripheral enhancements; they are the competencies that define what acute occupational therapy practice requires, and their absence represents a patient safety concern as well as an educational gap. Second, at regulatory level, the HPCSA minimum standards framework itself requires strengthening: the present study demonstrates empirically that regulatory compliance alone does not produce graduates who are competent in acute mental health practice. Addressing both levels, curriculum and regulatory, simultaneously is the only path to closing the preparation gap that this study has systematically documented.

## Author Contributions

P.Z.M. was the primary researcher responsible for the conceptualization of the study, data collection, data analysis, and writing of the manuscript. D.M.M. and D.N. were supervisors on the project who contributed to the conceptualization of the study, data analysis, consultations, and critical review of the manuscript.

## Funding

This work is based on the research supported in part by the National Research Foundation (NRF) of South Africa—Black Academics Advancement Program (GRANT NUMBER: BAAP2205036083).

## Conflicts of Interest

The authors declare no conflicts of interest.

## Data Availability

Data can be requested from the author(s).
